# How to measure temporal changes in care pathways for chronic diseases using health care registry data

**DOI:** 10.1186/s12911-019-0823-y

**Published:** 2019-05-30

**Authors:** Eugenio Ventimiglia, Mieke Van Hemelrijck, Lars Lindhagen, Pär Stattin, Hans Garmo

**Affiliations:** 1 Division of Experimental Oncology/Unit of Urology, IRCCS Ospedale San Raffaele, Milan, Italy; 2 King’s College London, School of Cancer and Pharmaceutical Sciences, Translational Oncology & Urology Research (Tour), 3rd Floor, Bermondsey Wing, Guy’s Hospital, London, SE1 9RT UK; 3 Uppsala Clinical Research Center, Uppsala, Sweden; 4 Department of Surgical Sciences, Uppsala University, Uppsala, Sweden; 5 Regional Cancer Centre, Uppsala Örebro, Uppsala, Sweden

**Keywords:** Ageing, Chronic disease, Prostate cancer, State transition

## Abstract

**Background:**

Disease trajectories for chronic diseases can span over several decades, with several time-dependent factors affecting treatment decisions. Thus, there is a need for long-term predictions of disease trajectories to inform patients and healthcare professionals on the long-term outcomes and provide information on the need of future health care. Here, we propose a state transition model to describe and predict disease trajectories up to 25 years after diagnosis in men with prostate cancer (PCa), as a proof of principle.

**Methods:**

States, state transitions, and transition probabilities were identified and estimated in Prostate Cancer data Base of Sweden (PCBaSe^Traject^), using nationwide population-based data from 118,743 men diagnosed with PCa. A state transition model in discrete time steps (i.e., 4 weeks) was developed and applied to capture all possible transitions (PCBaSe^Sim^). Transition probabilities were estimated for changes in both treatment and comorbidity. These models combined yielded parameter estimates to run an individual-level simulation based on the state-transition model to obtain prediction estimates. Predicted estimates were then compared to real world data in PCBaSe^Traject^.

**Results:**

PCBaSe^Sim^ estimates for the cumulative incidence of first and second transitions, death from PCa and death from other causes were compared to observed transitions in PCBaSe^Traject^. A good agreement was found between simulated and observed estimates.

**Conclusions:**

We developed a reliable and accurate simulation tool, PCBaSe^Sim^ that provides information on disease trajectories for subjects with a chronic disease on an individual and population-based level.

**Electronic supplementary material:**

The online version of this article (10.1186/s12911-019-0823-y) contains supplementary material, which is available to authorized users.

## Background

Many previously fatal diseases have become chronic diseases due to improvements in diagnosis and treatment [1]. These disease pathways (referred to as disease trajectories below) can span over several decades and incorporate a variety of treatments and outcomes. Moreover, ageing and increasing comorbidity will affect treatment decisions. Thus, there is a need to predict disease trajectories for several decades to inform patients and healthcare professionals on the long-term outcomes of disease and provide information on the need of future health care [2].

There is a wide range in the clinical course of prostate cancer (PCa) [3]. Due to improvements in detection, diagnostics, and treatment, many men currently diagnosed with PCa have a low risk of PCa death during the first 15 years after diagnosis and hence for many men PCa has become a chronic disease, albeit with a small risk of progression many years after date of diagnosis [4].

This study aimed to create a model to describe and predict disease trajectories over a period of 25 years using PCa as a proof of principle for a chronic disease. More specifically, we used data on PCa from nationwide, population-based health care registers in Sweden to predict disease trajectories in men with different disease severity, comorbidities, and treatments using state transition models.

## Methods

### Study population

This study was based on 118,743 men diagnosed with PCa from 1992 until 2014 who were registered in The National Prostate Cancer Register (NPCR) Sweden [5]. Men with unknown disease severity at time of diagnosis (3%) were excluded [6]. By use of the unique Swedish personal identity number, NPCR has been linked to other population-based healthcare registers and demographic databases to form the Prostate Cancer data Base Sweden (PCBaSe^Traject^). This provided additional information on cause of death, comorbidities, drug use, and socio-economic status, and treatment changes [7, 8]. Comorbidities were specifically measured with the Charlson comorbidity index (CCI), which was based on discharge diagnoses retrieved from the National Patient Register and the National Cancer Register [9]. The linkage of PCBaSe was approved by the Research Ethics Board at Umeå University.

The men were divided into five groups based on the primary management strategy: active surveillance (AS), radical prostatectomy (RP), radiotherapy (RT) performed as either external beam radio therapy or brachy therapy, watchful waiting (WW), and androgen deprivation therapy (ADT). Different risk categories were defined for each management strategy. For AS risk categories, we applied a modification of the National Comprehensive Cancer Network guidelines [10]. The two lowest NCCN risk categories (low and intermediate-risk PCa) were divided into two subgroups each. The least favourable intermediate-risk category was not considered as suitable for AS, resulting in three AS risk categories (Additional file 2: Table S1). Curative treatment consisted of RP or RT, which could be either primary treatment or initiated after a period of AS, i.e. deferred RP or RT. Primary and deferred RPs were divided into six risk categories based on pathological tumour stage (pT stage) and pathological Gleason Grade Group (pGGG) as recorded in NPCR (Additional file 2: Table S2). Primary and deferred RT was divided into eight risk categories (Additional file 2: Table S3). WW as primary management strategy was classified into six risk categories, based on T stage, N stage, M stage, serum levels of prostate-specific antigen (PSA), and Gleason Grade Group (GGG) (Additional file 2: Table S4).

Men receiving ADT were divided into eight risk categories (Additional file 2: Table S5). ADT consisted of either antiandrogen monotherapy (AA) or gonadotropin-releasing hormone (GnRH) agonists (Additional file 2: Figure S1). For all treatment-specific risk categories, the lowest category indicated men with the most favourable risk.

### Missing data

There is no specific follow-up data in NPCR. For example, men primarily managed with AS who eventually experience disease progression can be treated with curative intention with RP or RT. At this point, the updated TNM stage, GGG and PSA have not routinely been reported to NPCR. This yields a missing data problem. We solved this problem by applying multiple imputation by chained equations [11] using five imputation datasets. Similar problems with lack of detailed disease progression data also appeared for other non-primary treatments, where change was triggered by disease progression and these situations were also solved by multiple imputation. Further details regarding imputation models are presented in the Additional file 1.

### Analysis

**Fig. 1:**
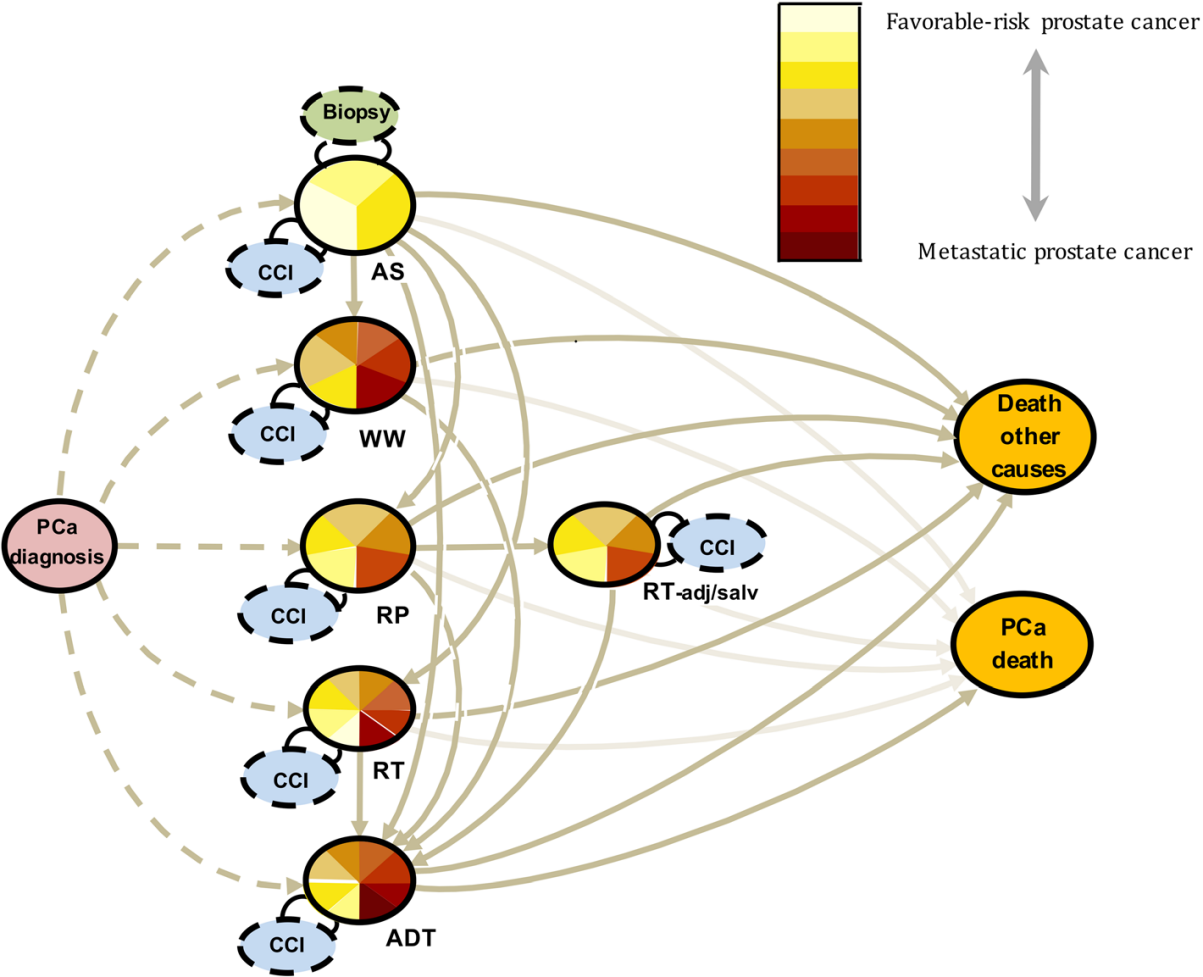
States and state transitions in Prostate Cancer data Base of Sweden^Sim^ (PCBaSe^Sim^). State transition model of transitions (arrows) between states (circles) for men diagnosed with prostate cancer. The states are active surveillance (AS), watchful waiting (WW), curative treatment; radical prostatectomy (RP) or radiotherapy (RT), adjuvant or salvage radiotherapy following RP (RT-adj/salv), androgen deprivation therapy (ADT), death from other causes, and prostate cancer death. Dashed lines represent the choice of primary treatment following diagnosis (not part of the model), solid lines are transitions included in the models. Multi-colored circles represent transient states with colors indicating the proportion of men with increasing disease risk categories defined by data at date of diagnosis. Orange circles represent absorbing states. Dashed circles represent additional information gathered to facilitate estimates of transition probabilities, i.e. biopsy and Charlson Comorbidity Index (CCI). Risk categories are defined in detail in the Additional file 1

We applied a method based on a state transition model (Fig. 1). Men diagnosed with PCa entered their primary state according to their primary treatment (AS, RP, RT, WW, ADT) and their treatment specific risk category (AS_1_-AS_3_, RP_1_-RP_6_, RT_1_-RT_8_, WW_1_-WW_6_, and ADT_1_-ADT_8_). The probability of a transition to another state was based on age, comorbidity, history of previous treatments, and treatment-specific risk category. All transitions were considered irreversible and state transitions were allowed until an absorbing state, i.e. PCa-death or death from other causes, was reached. Transitions between treatment-specific risk categories were not considered, i.e. each man stayed in his designated treatment risk category until a new treatment was introduced. Our model building consisted of several steps:

First, we randomly split our set of data in two equally large subsets, the first set was a training set that was used to estimate transition probabilities and the second set was used to assess internal validity of the simulation process.

Second, we simplified follow-up time by use of four-week time steps. At the end of each time step a man either remained in his current state or transited to a new state. The discretised follow-up data was arranged using long format, i.e., each man was represented by several rows of data, one for each time step in which he was alive. Age and CCI were updated in each time step.

Next, we estimated state transition probabilities, which were determined from both registered and non-registered data regarding date of treatment change. Specifically, for all treatment state transitions, except the transition from AS ➔ WW, the date of treatment change was retrieved from PCBaSe^Traject^ using the training set of data. Left truncation on January 1, 2006, was used as previously described [8]. The non-registered transition date for the transition of AS ➔ WW was managed as previously described [12] (Additional file 2: Figures S2 and S3). We considered a change in CCI as a state transition. For such a CCI transition, we applied a CCI state transition model as previously described [9]. The probability of CCI changes was state-specific, i.e. different CCI model parameters were estimated for each state.

Finally, our prediction was carried out as a microsimulation, an individual-level simulation based on the state-transition model [13]. Simulations were performed for each specific combination of treatment, age and CCI at treatment start, and treatment-specific risk categories. The simulation process consisted of three different steps, which are described in brief below. Further details and specifications can be found in the Additional file 1.

1. A man’s vital status at the end of each time step was determined as alive, dead from PCa or dead from other causes. The probability of PCa death was modelled in a logistic regression, whereas the probability of death from other causes was modelled conditioned on no previous PCa death in a second logistic regression (see Additional file 1 for further details).
2. For a man who was alive at the end of a time step, we determined if a treatment change had occurred. Such change could be either direct, when the treatment risk category in the new state was known and remained unchanged, e.g. RP ➔ RT_adj/salv_, or preceded by an estimation of new treatment-specific risk category (Additional file 2: Table S6). In the latter case, an ordinal regression analysis determined the new risk category, at the same time step as the transition.
3. If a man was alive at the end of a time step, CCI was updated, according to a separate CCI transition model based on a combination of Poisson regression and logistic regression models, as described previously [9]. The CCI transition model was specific for each individual treatment.

We refer to this simulation method in the following text as PCBaSe^Sim^. Further specifications of possible transitions and used models are provided in the Additional file 1. In order to assess internal validity of PCBaSe^Sim^, we graphically compared cumulative incidence of subsequent transitions obtained from the simulation with those observed in validation set.

## Results

The study population consisted of 118,743 men diagnosed with PCa and included in NPCR. Most of these men (*n* = 32,537, 27%) were primarily managed with RP (Table 1). Detailed characteristics of men included in PCBaSe^Traject^ were previously described [7, 8].

**Table 1:** Baseline characteristics for men in Prostate Cancer data Base Sweden (PCBaSe)^Traject^

	Deferred treatment as part of AS ➔ WW model (*n* = 23,649)		Deferred treatment WW (*n* = 7286)		Radical prostatectomy RP (n = 32,537)		Radio therapy RT (*n* = 19,019)		Anti-androgen monotherapy AA (*n* = 7178)		Gonadotropin releasing hormone agonists GnRH (*n* = 29,074)		Total (*n* = 118,743)	
Age, median (Q1-Q3)	69	(64–74)	77	(73–81)	63	(59–67)	67	(62–71)	76	(71–81)	77	(71–82)	69	(63–76)
Age, n (%)														
≤ 55	1008	(4.3)	33	(0.5)	4120	(12.7)	875	(4.6)	55	(0.8)	341	(1.2)	6432	(5.4)
56–60	2332	(9.9)	116	(1.6)	6942	(21.3)	2403	(12.6)	153	(2.1)	803	(2.8)	12,749	(10.7)
61–65	4543	(19.2)	296	(4.1)	10,356	(31.8)	4775	(25.1)	475	(6.6)	1959	(6.7)	22,404	(18.9)
66–70	6172	(26.1)	800	(11.0)	8635	(26.5)	6053	(31.8)	964	(13.4)	3535	(12.2)	26,159	(22.0)
71–80	8241	(34.8)	3929	(53.9)	2461	(7.6)	4880	(25.7)	3734	(52.0)	13,167	(45.3)	36,412	(30.7)
81+	1353	(5.7)	2112	(29.0)	23	(0.1)	33	(0.2)	1797	(25.0)	9266	(31.9)	14,584	(12.3)
Year of diagnosis, n (%)														
1992–1997	558	(2.4)	243	(3.3)	513	(1.6)	245	(1.3)	22	(0.3)	561	(1.9)	2142	(1.8)
1998–2004	4472	(18.9)	2144	(29.4)	7113	(21.9)	5373	(28.3)	1024	(14.3)	8672	(29.8)	28,798	(24.3)
2005–2008	5770	(24.4)	2369	(32.5)	9363	(28.8)	4894	(25.7)	1742	(24.3)	9353	(32.2)	33,491	(28.2)
2009–2011	5969	(25.2)	1679	(23.0)	7911	(24.3)	4804	(25.3)	1841	(25.6)	5893	(20.3)	28,097	(23.7)
2012–2014	6880	(29.1)	851	(11.7)	7637	(23.5)	3703	(19.5)	2549	(35.5)	4595	(15.8)	26,215	(22.1)
T stage, n (%)														
T1a	2564	(10.8)	440	(6.0)	276	(0.8)	99	(0.5)	25	(0.3)	129	(0.4)	3533	(3.0)
T1b	963	(4.1)	499	(6.8)	247	(0.8)	194	(1.0)	101	(1.4)	365	(1.3)	2369	(2.0)
T1c	15,243	(64.5)	2373	(32.6)	20,010	(61.5)	7496	(39.4)	1580	(22.0)	3559	(12.2)	50,261	(42.3)
T2	4738	(20.0)	2464	(33.8)	10,881	(33.4)	7201	(37.9)	2659	(37.0)	8927	(30.7)	36,870	(31.1)
T3	0	(0.0)	1362	(18.7)	979	(3.0)	3883	(20.4)	2436	(33.9)	12,517	(43.1)	21,177	(17.8)
T4	0	(0.0)	49	(0.7)	12	(0.0)	71	(0.4)	280	(3.9)	3002	(10.3)	3414	(2.9)
TX/Missing	141	(0.6)	99	(1.4)	132	(0.4)	75	(0.4)	97	(1.4)	575	(2.0)	1119	(0.9)
N stage, n (%)														
N0	1831	(7.7)	292	(4.0)	7472	(23.0)	6004	(31.6)	766	(10.7)	1418	(4.9)	17,783	(15.0)
N1	0	(0.0)	44	(0.6)	278	(0.9)	317	(1.7)	321	(4.5)	1627	(5.6)	2587	(2.2)
NX	21,818	(92.3)	6950	(95.4)	24,787	(76.2)	12,698	(66.8)	6091	(84.9)	26,029	(89.5)	98,373	(82.8)
Gleason Grade Group /WHO, n (%)														
GGG1	20,019	(84.7)	2860	(39.3)	16,783	(51.6)	6653	(35.0)	1181	(16.5)	3226	(11.1)	50,722	(42.7)
GGG2	2295	(9.7)	1469	(20.2)	8564	(26.3)	4750	(25.0)	1537	(21.4)	3398	(11.7)	22,013	(18.5)
GGG3			969	(13.3)	3291	(10.1)	3007	(15.8)	1476	(20.6)	4209	(14.5)	12,952	(10.9)
GGG2–3	202	(0.9)	257	(3.5)	790	(2.4)	388	(2.0)	168	(2.3)	1331	(4.6)	3136	(2.6)
GGG4			662	(9.1)	1792	(5.5)	2025	(10.6)	1321	(18.4)	6155	(21.2)	11,955	(10.1)
GGG5			202	(2.8)	687	(2.1)	1496	(7.9)	1028	(14.3)	6874	(23.6)	10,287	(8.7)
Only WHO-grade	1096	(4.6)	740	(10.2)	600	(1.8)	671	(3.5)	315	(4.4)	3055	(10.5)	6477	(5.5)
Missing	37	(0.2)	127	(1.7)	30	(0.1)	29	(0.2)	152	(2.1)	826	(2.8)	1201	(1.0)
PSA, median (Q1-Q3)	6.0	(4.2–8.2)	18	(12–26)	6.8	(4.8–10)	10	(6.4–18)	22	(12–47)	45	(19–139)	9.9	(5.8–24)
Mode of detection, n (%)														
Screening^f^	10,024	(42.4)	1842	(25.3)	17,060	(52.4)	8119	(42.7)	2020	(28.1)	3912	(13.5)	42,977	(36.2)
LUTS	7705	(32.6)	2572	(35.3)	7453	(22.9)	5133	(27.0)	3134	(43.7)	12,024	(41.4)	38,021	(32.0)
Other symptoms	4258	(18.0)	2127	(29.2)	5606	(17.2)	4244	(22.3)	1743	(24.3)	10,962	(37.7)	28,940	(24.4)
Missing	1662	(7.0)	745	(10.2)	2418	(7.4)	1523	(8.0)	281	(3.9)	2176	(7.5)	8805	(7.4)
CCI, n (%)														
0	15,370	(65.0)	4257	(58.4)	25,259	(77.6)	13,369	(70.3)	4275	(59.6)	16,427	(56.5)	78,957	(66.5)
1	4228	(17.9)	1435	(19.7)	3955	(12.2)	3172	(16.7)	1461	(20.4)	6179	(21.3)	20,430	(17.2)
2	2356	(10.0)	945	(13.0)	1971	(6.1)	1511	(7.9)	806	(11.2)	3737	(12.9)	11,326	(9.5)
3+	1695	(7.2)	649	(8.9)	1352	(4.2)	967	(5.1)	636	(8.9)	2731	(9.4)	8030	(6.8)
Risk category, n (%)														
1	7546^a^	(31.9)	1001^b^	‘(13.7)	8985^c^	(27.6)	3735^d^	(19.6)	922^e^	(12.8)	6854^e^	(23.6)		
2	13,515^a^	(57.1)	1004^b^	‘(13.8)	9997^c^	(30.7)	1563^d^	(8.2)	870^e^	(12.1)	2585^e^	(8.9)		
3	2588^a^	(10.9)	947^b^	‘(13.0)	5094^c^	(15.7)	2285^d^	(12.0)	1495^e^	(20.8)	2855^e^	(9.8)		
4			1307^b^	‘(17.9)	5893^c^	(18.1)	2805^d^	(14.7)	1162^e^	(16.2)	1811^e^	(6.2)		
5			1281^b^	‘(17.6)	2480^c^	(7.6)	1844^d^	(9.7)	1007^e^	(14.0)	3605^e^	(12.4)		
6			1746^b^	‘(24.0)	88^c^	(0.3)	2146^d^	(11.3)	634^e^	(8.8)	2468^e^	(8.5)		
7							2658^d^	(14.0)	492^e^	(6.9)	1231^e^	(4.2)		
8							1983^d^	(10.4)	596^e^	(8.3)	7665^e^	(26.4)		

*CCI* Charlson comorbidity index, *LUTS* lower urinary tract symptoms

^a^Risk categories AS_1_- AS_3_

^b^Risk categories WW_1_-WW_6_

^c^Risk categories RP_1_-RP_6_

^d^Risk categories RT_1_-RT_8_

^e^Risk categories ADT_1_-ADT_8_

^f^Screening, prostate cancer detected due to work-up after PSA testing in asymptomatic men

Our proposed model starts at time of PCa diagnosis and ends in either of the following absorbing states: death from PCa or death from other causes. Figure 1 illustrates states and state transitions, with all the possible treatment changes included in the model.

**Fig. 2:**
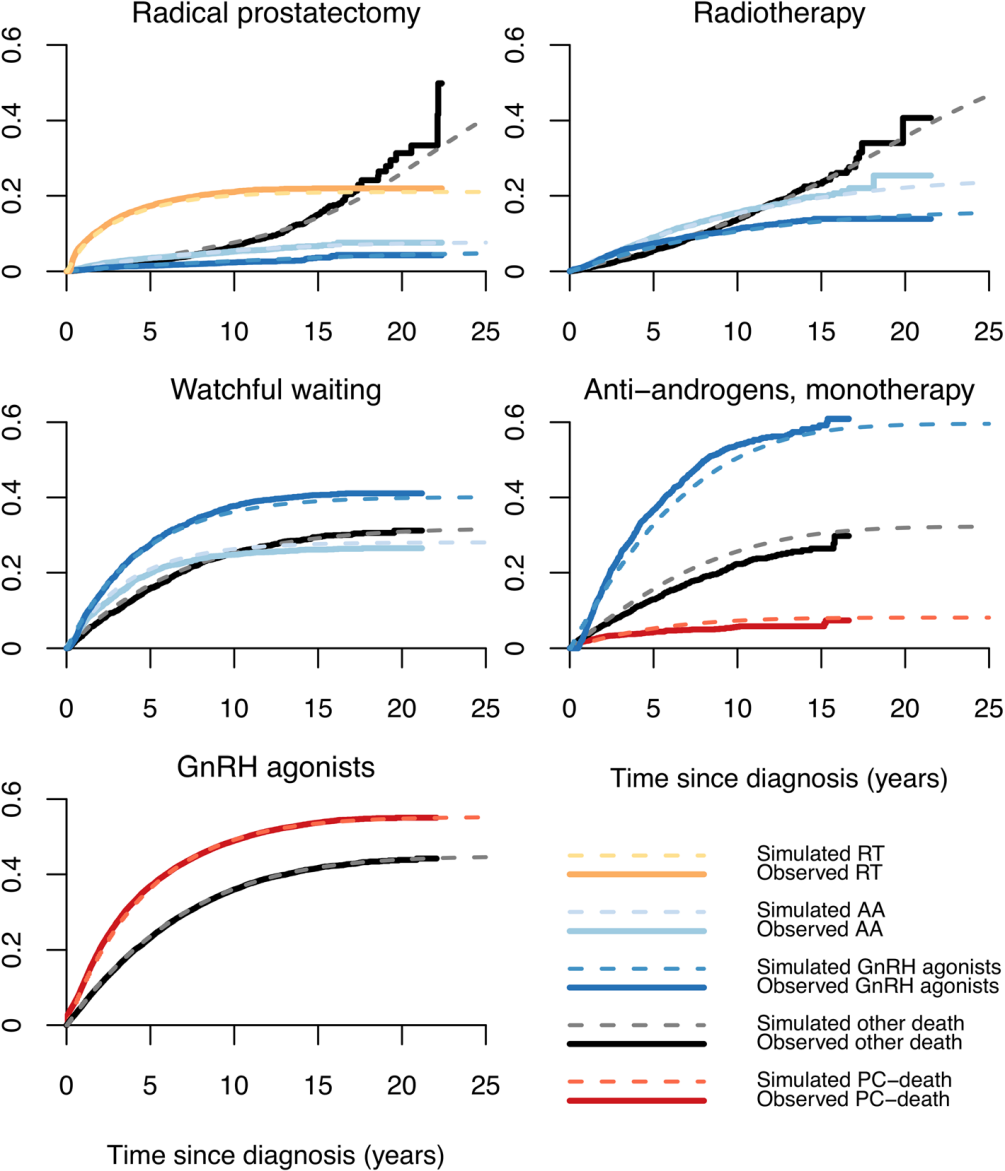
Cumulative incidence of first observed and simulated transition according to primary management strategy in men with prostate cancer in PCBaSe. Graphs show the cumulative incidence of first observed transition in PCBaSe^Traject^ (continuous line) compared to the cumulative incidence of first simulated transition in PCBaSe^Sim^ (dashed lines). PCBaSe^Sim^ transitions are based on the simulation of men in PCBaSe^Traject^ stratified by primary management strategy. AA: anti-androgens; GnRH: gonadotropin releasing hormone agonists; PC: prostate cancer; RP: radical prostatectomy; RT: radiotherapy; WW: watchful waiting

To assess the internal validity of the simulation, the cumulative incidence of the first transitions observed in PCBaSe^Traject^ was compared with the simulated first transitions for the same men up to 25 years after diagnosis. To decrease random error, we ran the simulation 100 times for each man. Observed and simulated first transitions almost perfectly overlapped for all groups (Fig. 2). At ten years after RP, 21% of the observed PCBaSe^Traject^ cohort had transitioned to adjuvant/salvage radiotherapy vs. 20% as predicted by PCBaSe^Sim^. Estimates at 20-years were still consistent for all the analysed primary management strategies (e.g., 5% of men treated with primary RP received GnRH agonists in both PCBaSe^Traject^ and PCBaSe^Sim^).

**Fig. 3:**
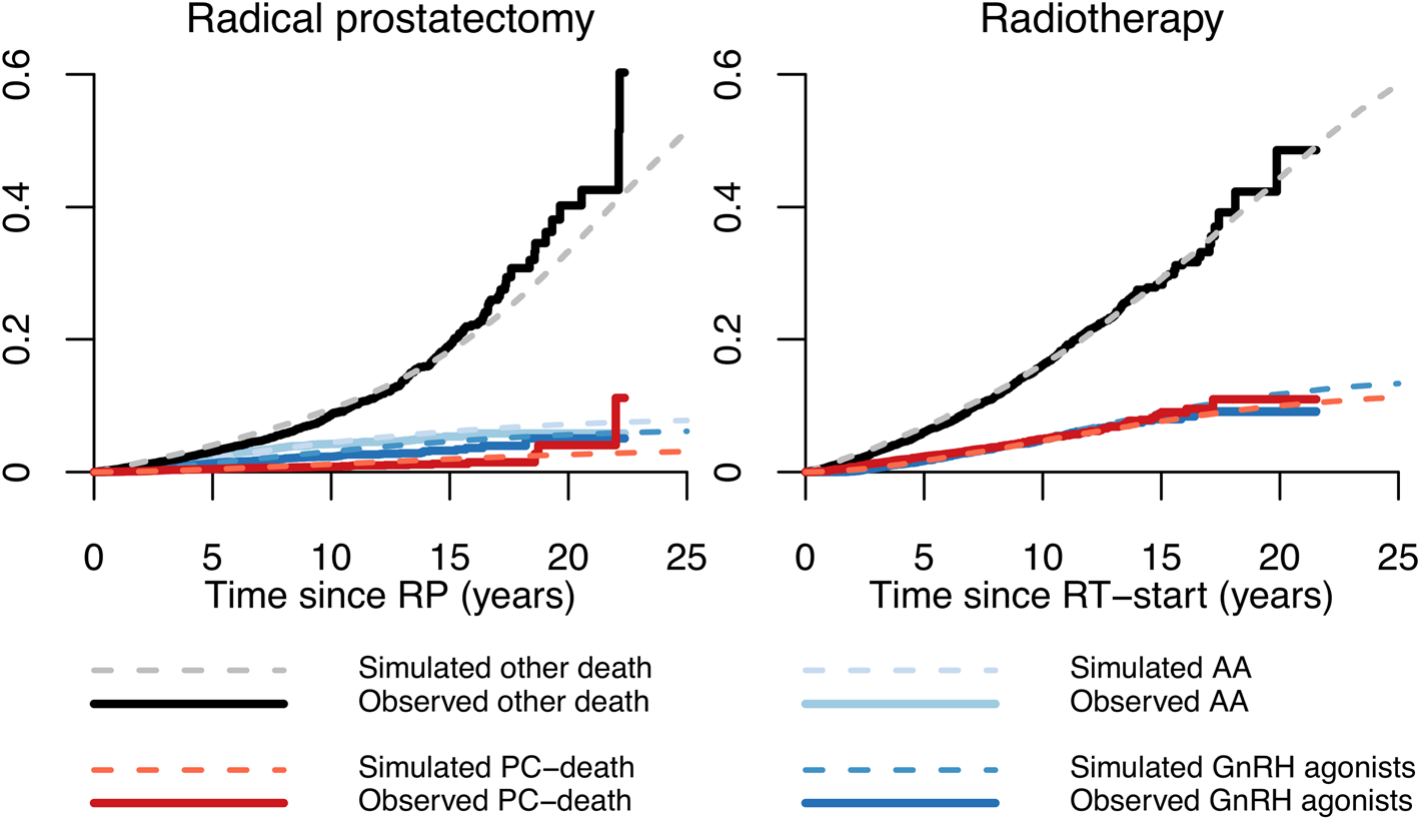
Cumulative incidence of second observed and simulated transition according to primary management strategy in men diagnosed with prostate cancer and primarily treated with radical prostatectomy and radiotherapy. Graphs show the cumulative incidence of second observed transition in PCBaSe^Traject^ (continuous line) compared to the cumulative incidence of second simulated transition in PCBaSe^Sim^ (dashed lines). PCBaSe^Sim^ transitions are based on the simulation of men in PCBaSe^Traject^ primarily treated with radical prostatectomy and radiotherapy. AA: anti-androgens; GnRH: gonadotropin releasing hormone agonists; PC: prostate cancer; RP: radical prostatectomy; RT: radiotherapy

**Fig. 4:**
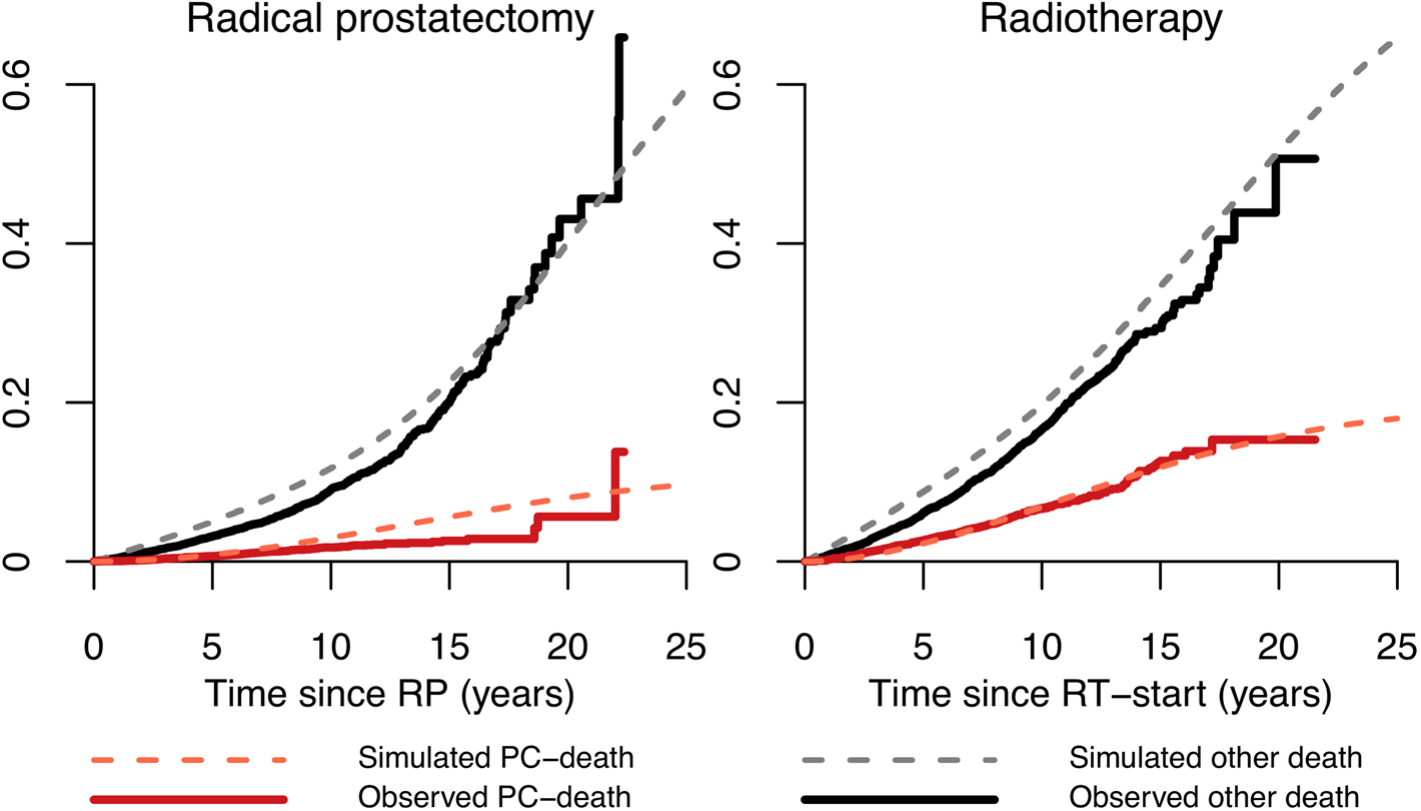
Prostate cancer death and death from other causes in PCBaSe^Traject^ and PCBaSe^Sim^. Graphs show the cumulative incidence of prostate cancer death and death from other causes (i.e. final absorbing states) in PCBaSe^Traject^ (observed, continuous line) compared to PCBaSe^Sim^ (simulated, dashed lines). PCBaSe^Sim^ transitions are based on the simulation of men in PCBaSe^Traject^

Similarly, we analysed second transitions. Men first treated with RP or RT, which are the most common treatment strategies overall for PCa, were followed until their second transition. Also in this model, the observed and simulated cumulative incidences were very similar, e.g. 10-year proportion of men treated with primary RT and later receiving GnRH agonists was 5% in both PCBaSe^Traject^ and PCBaSe^Sim^. At 15 years these proportions were 7% in PCBaSe^Traject^ and 9% in PCBaSe^Sim^, respectively (Fig. 3**)**. Eventually, the agreement between predicted vs. observed cumulative incidence of death was acceptable for both PCa and other causes of death (Fig. 4).

## Discussion

We developed, applied, and tested a novel state transition model, based on longitudinal data in health care registers including men with PCa, to predict long term disease trajectories. More specifically, we tested the accuracy of first and second transitions (i.e. treatment changes), as well as the transitions to final absorbing states, by comparing observed data vs. data obtained by our newly developed tool, PCBaSe^Sim^.

Treatment decisions are a challenging process in real life, as well as predictions of health-related outcomes. Due to the long life expectancy, e.g. > 20 years for healthy men with low-risk PCa [4], and the availability of many therapeutic options [3], PCa disease trajectories can thus be difficult to predict at time of diagnosis. These pathways include different treatment options and may be protracted over time. Although international guidelines recommend therapeutic options according to disease status, there are several combinations of disease states and life expectancy for which there is little evidence to support treatment decisions [3]. Moreover, recently introduced management strategies, e.g. active surveillance, lack long-termfollow-up, so there are no data on which to base outcome predictions. Previous attempts have been made to implement state-transition models in urology [14, 15] and other medical areas [16], mainly from the health-economy standpoint. However, these attempts were not based on a detailed and comprehensive, population-based source of data, such as PCBaSe^Traject^. Therefore, previous models have not been validated against real world data. In contrast, the state transition model we propose in this paper was compared with real world data, and provides as a user-friendly data output that makes both interpretation of results and replicability easy.

More specifically, these results provide a proof-of-principle for state transition models that can be applied to many other chronic diseases for which there is also a need for long term predictions of outcomes. Using complete nationwide, population-based data of ~ 120,000 Swedish men with PCa [8], we were able to develop a simulation programme, PCBaSe^Sim^, which reliably models treatment changes up until 25 years after diagnosis. To assess the reliability of our state transition model, we compared real-worldfollow-up data in PCBaSe^Traject^ with simulated data from PCBaSe^Sim^. We found that observed and simulated transition profiles were overlapping and consistent, suggesting that PCBaSe^Sim^ estimates were accurate and reliable. Moreover, in PCBaSe^Sim^, it is possible to set baseline parameter (e.g. number of subjects, initial state, subject- and disease-specific features, temporal duration of the simulation) prior to starting the simulation process. During the simulation, individual features are constantly updated (e.g. age increase, comorbidity changes [9]) and used for estimation of transition probabilities. Once a simulation is complete (i.e. end of virtual follow-up time), it is possible to retrieve every change in both treatment and individual characteristics (i.e. age, CCI, and risk group).

Our proposed state transition modelling approach consistently differs from conventional survival analyses and prediction tools, which focus on a single outcome over time, with little information regarding the actual path that leads to a specific outcome and the changes in the disease trajectory [17]. Conventional survival analyses inevitably result in a consistent loss of information and accuracy, especially when dealing with extended follow-up times. We have previously shown that it is possible to model temporal changes in comorbidity for cancer patients [9]. In PCBaSe^Sim^, thanks to the continuous update of baseline characteristics at the end of every time step, not only are the predictions likely to be accurate, but also is it possible to keep track of individual and disease-specific features and trajectories. Although the difference in model fit between time-updated CCI and CCI only measured at baseline was small (data not shown), we used the time-updated CCI since this approach was crucial to solve the problem of non-registered AS ➔ WW transition [12]. Therefore, in order to be congruent with our previous publication [12] as well as with future studies on health economy, we support the use of time-updated CCI since increases in comorbidity are related to increased costs and therefore affect treatment decisions.

Moreover, PCBaSe^Sim^ makes it possible to model disease trajectories over a long time-horizon and can therefore make long-term predictions even in the absence of “observed” (i.e. registered) data. Eventually, the flexibility of the model allows to include novel modelling parameters and states.

The main limitation of our proposed model is the absence of an external validation. However, PCBaSe^Sim^ is ready to be tested in an external setting, and the authors welcome collaborators to validate our simulation program. It is worth noticing that in PCBaSe^Traject^, we had to use left truncation on January 1, 2006 since the Swedish Prescribed Drug Registry was initiated on July 1, 2005 [8]. The relatively low number of diagnosis during the early PCBaSe^Rapid^ period is caused by this left truncation. This could theoretically limit the predictive power of PCBaSe^Sim^. On the other hand, we argue that the most recent data are the most valuable for prediction for current patients and that therefore it is advantageous that the impact of historically older diagnosis and treatments on predictions was limited by the use of this approach. Further limitations include the use of administrative data for the definition of comorbidities, though it has previously been shown that the accuracy of ICD codes for discharge diagnoses in the Patient Registry is high in the range of 85–95% [18]. Another limitation is represented by missing data related on some transitions/risk categories. However, we have previously [12] and in this current study provided reliable solutions to these issues.

## Conclusion

We developed a reliable and accurate simulation tool, PCBaSe^Sim^, to provide information to patients and healthcare professionals, on an individual as well as population-based level on treatment trajectories. Aside from clinical implications, our model provides information applicable to healthcare resources allocation. Since it is possible to keep track of specific disease trajectories including the mean time spent in each state, estimates for costs can be obtained by use of our model.

## Additional files

Additional file 1: Detailed specifications of the model, including states, transitions, and transition probabilities. (DOCX 45 kb)

Additional file 2: Supplementary figures and tables. (DOCX 212 kb)

## Data Availability

Data used for the current study can be retrieved by contacting hans.garmo@kcl.ac.uk. The steering groups of NPCR and PCBaSe welcome external collaborations. For more information please see www.npcr.se/in-english where registration forms, manuals, and annual reports from NPCR are found as well as a full list of publications from PCBaSe.

## Abbreviations

AA: Anti-androgens
ADT: Androgen deprivation therapy
AS: Active surveillance
CCI: Charlson comorbidity index
GGG: Gleason grading group
GnRH: Gonadotropin releasing hormone
NPCR: National Prostate Cancer Register
PCa: Prostate cancer
PCBaSe: Prostate Cancer database of Sweden
RP: Radical prostatectomy
RT: Radiotherapy
WW: Watchful waiting
