# Supplementary material for: How to measure temporal changes in care pathways for chronic diseases using health care registry data
Source: BMC Med Inform Decis Mak. 2019 May 30;19:103. doi: 10.1186/s12911-019-0823-y (PMC6543619; doi:10.1186/s12911-019-0823-y)
Supplement: Supplementary file 1 — Detailed specifications of the model, including states, transitions, and transition probabilities. (DOCX 45 kb) [file 12911_2019_823_MOESM1_ESM.docx]

**APPENDIX: Specifications of state transition model**

We used a state transition model in which we estimated the probability of changes in treatment strategy (“transition probabilities”) between pre-defined treatment strategies (“states”), as illustrated in Figure 1. The arrows (transitions) between circles (states) correspond to changes in treatment. The colours of the states correspond to the management strategy risk categories described above. Details and model specifications are presented below.

1. **Definition of states, transition probabilities, and predictions**
   1. *States*

Figure 1 illustrates the transitions that are the basis for our study. The states are either initial states, transition states (multi coloured circles for both) or absorbing states (orange circles). The blue and green circles represent additional information obtained to facilitate the estimation of transition probabilities and describe possible transitions back to the same state. The arrows (transitions) between circles (states) correspond to treatment changes.

WW is an important secondary state for men who start their PCa pathway in AS, but this transition cannot be defined by use of data in PCBaSe, and is therefore denoted as a *non-registered state transition.* Our solution to the non-registered state transitions was recently described [12].

In short, our solution is based on balancing the flow of men reaching ADT starting in WW and starting in AS. It involves information on biopsies (green circle, i.e. a proxy measure of cancer progression) and comorbidity as measured by CCI (blue circles) and divides the problem into two types based on initial risk category. In both types, we allowed for the transition AS🡪 WW 🡪 ADT as displayed in **Supplementary** **Figure** **2**. For AS given to men in the lowest AS risk group (AS_1_, **Supplementary** **Table 1**) we also allowed for a direct transition from AS to ADT. This direct ADT transition was denoted AS-failure and we did not consider such transitions for men in the intermediate AS risk group (AS_2_) and highest AS risk group (AS_3_) (**Supplementary** **Figure 2**). In addition to the absorbing states PCa death and death from other causes, our solution previously presented [12] considered additional states (RP/RT, ADT, and AS-failure) to be absorbing, as displayed in **Supplementary** **Figure 2**, to facilitate the modelling of AS 🡪 WW transitions. However, during the simulation process of PCBaSe^Sim^ these states were not considered absorbing.

WW is a treatment strategy not only for men with risk category AS_1_-AS_3_ but also for men with more severe prostate cancer. To allow for WW, we consider WW as two different states WW_AS_ and WW_Non-AS_ as displayed in (**Supplementary** **Figure 3**). Therefore, our WW state in Figure 1 is a simplified compared to the actual modelling of the WW state.

We also made a simplification for the ADT state (**Figure 1**). The choice of ADT is related to risk of PCa death and the rate of change in CCI. Therefore, we split the ADT state into two transient states: Anti-androgens (AA) and Gonadotropin-releasing Hormone (GnRH) agonists, with transitions shown in **Supplementary Figure 1**. Below we present models for the AA and GnRH states separately.

- 1. *Transition probabilities*

Men either remained in a state or changed states in discrete time steps of Δ*t* = 28 days. A complete list of models used to estimate transition probabilities is presented below and **Supplementary** **Table 6**.

- 1. *Predictions*

Once the transition probabilities had been estimated, predictions were carried out using microsimulation, an individual-level simulation based on the state-transition model ([4](#_ENREF_4)). It is possible to perform simulation for each specific combination of treatment, age, and CCI at start of treatment, and treatment specific risk category. For simulations for men on AS, we also allowed the PSA level to be specified (in the range of the specific risk category). For the specific purpose of assessing internal validity, in each simulation we replicated 100 times each man with identical baseline characteristics to those observed in real data, PCBaSe^Traject^ (20 for each imputation dataset) to achieve good precision in estimates. Simulations were up to 25 years. In all simulations, CCI was updated using a separate CCI simulation as described previously [9].

As disease risk category was an important covariate in all transitions, it needs to be noted that data in NPCR is mainly collected in the initial phase of the disease. Therefore, the determination of treatment specific risk categories involves a missing data problem that was solved for each treatment state transition. We applied multiple imputation by chained equations [11] using five imputation dataset to handle missing data problems for disease severity in adjacent states following the initial one.

1. **Statistical methods to obtain transition probabilities**
   1. *Non-observable state transition*

Our solution has been described previously [12] and, shortly, above.

- 1. *Observable state transitions*

Probabilities for observable transitions were retrieved using standard statistical software tools, including standard techniques for handling multiple imputation. We considered two types of death: PCa-death and death from other causes (referred to as ‘Death’ in the below). All observable state transitions are detailed below:

**Transitions to death from other causes**

The absorbing state Death could be reached from all states in the model.

(Any state 🡪 Death)

The risk of death from other causes was dependent on age, Charlson Comorbidity Index (CCI), interaction between age and CCI, current state, and interaction between current state/time after curative treatment or ADT/age/CCI. Men selected for curative treatment are healthier compared to the background population [9,19], so that we modelled these covariates to accommodate this selection. The probability of death was modelled in a logistic regression. Details on how covariates were handled in the model can be found in **Supplementary** **Table 6**.

**Transitions to PCa-death**

For men in the states AS, WW, RP, and RT, the direct transition to PCa death is very rare (<0.5% of deaths in PCBaSe^Traject^), as ADT is usually initiated prior to PCa death. This is indicated by the light grey arrows in **Figure 1**. Instead of modelling these rare transitions, we made an assumption that GnRH agonists had been initiated in these men one month prior to PCa death. We dealt with these few extra GnRH cases in the model for the transition GnRH 🡪 PC-death. Therefore, the absorbing state PCa death could only be reached from the ADT state.

(AA🡪 PCa-death)

This transition was modelled in a logistic regression involving age, CCI, AA risk category and treatment path prior to reaching the AA state.

(GnRH🡪 PCa-death)

This transition was modelled in a logistic regression involving age, CCI, GnRH-risk category, time spent in the GnRH state, and time spent in GnRH state ≤28 days. The latter interaction terms were introduced to account for the artificial assumption of GnRH introduced a month prior to PCa death described above.

**Transitions to GnRH**

This transient state could be reached from all other transient states. The models for transitions to the GnRH state were all conditioned on no previous death (neither from PCa death nor death from other causes).

As the risk of PCa death for a GnRH treated man depends on disease severity, we accounted for this by introducing eight GnRH-risk categories (**Supplementary** **Table 5)**. These categories were known for all men for whom GnRH was the primary treatment in PCBaSe^Traject^ (n=32,589). GnRH-risk categories were imputed for men entering the GnRH state:

1. directly following AS (n=1,267),
2. following RP in combination with any other treatment (n=1,293)
3. following RT in combination with any other treatment (n=1,935)
4. directly following WW (n=1,910)
5. following AA (n=1,768)

The imputation was done in separate imputations a)-e) where information regarding GnRH risk categories from the men initially treated with GnRH was combined with the information retrieved for the men in groups a)-e), using multiple imputation with chained equations [11]. Imputations were based on age, time to death and cause of death, time to first CCI-change and size of first CCI change. For men starting on AA, the GnRH-risk was never allowed to be lower than their AA-risk category. For men entering the GnRH-state directly from AS or WW, the GnRH-risk was never lower than indicated by the TNM-stage, PSA-level and Gleason grade group at date of diagnosis.

Based on the imputation datasets, a GnRH-risk category was determined for each man reaching the state GnRH by considering the entrance to the GnRH-state as a two-step procedure: entering the state and determination of GnRH-risk category (GnRH 🡪 GnRH-risk). Men reaching the states ADT (following AS) and AS-failure of **Supplementary** **Figure 2** may have undergone a transition to GnRH. For these scenarios, we did not make a difference between the ADT and AS-failure states.

(ADT/AS-failure 🡪 GnRH)

A logistic regression model was applied to all men reaching the states ADT or AS-failure. This model determined if entering ADT/AS-failure meant entering the GnRH-state or the AA-state. The details for the logistic regression model are specified in **Supplementary** **Table 6**.

(RP 🡪 GnRH)

The transition probabilities for RP 🡪 GnRH were determined with a logistic regression involving age, CCI, RP-risk category, time spent in the RP state and treatment pathway prior to RP state. Further details are specified in **Supplementary** **Table 6**.

(RT 🡪 GnRH)

This transition was handled with a logistic regression involving age, CCI, RT-risk category, time spent in the RT state and treatment pathway prior to RT state. Further details are specified in **Supplementary** **Table 6**.

(RT_adj/salv_ 🡪 GnRH)

This transition was handled using logistic regression involving age, CCI, RP-risk category, time spent in the RT_adj/salv_-state, and treatment pathway prior to RT_adj/salv_ state. The model is further specified in **Supplementary** **Table 6**.

(WW 🡪 GnRH)

Since this is an observable transition for men starting in WW, we used a logistic regression involving age, CCI, WW-risk category, and time spent in the WW-state. This model was used for men in risk categories WW_1_-WW_6_, whereas men in risk categories AS_1_-AS_3_ were handled in accordance with our solution for the non-observable transition, as previously described [12]. For further details see **T Supplementary** **Table 6**.

(AA 🡪 GnRH)

This transition was determined with a logistic regression similar to the WW 🡪 GnRH described above. Further details are specified in **Supplementary** **Table 6**.

(GnRH 🡪 GnRH-risk)

Imputed GnRH-risk categories are displayed in **Supplementary** **Table** **6**. Ordinal regression models and imputation were used to estimate the probabilities of entering the different GnRH-risk categories. The ordinal regression model is specified in **Supplementary** **Table 6**.

**Transitions to AA**

This transient state could be reached from all other states, except the GnRH-state. This state transition has much in common with the transitions to GnRH. The major difference is that we also conditioned on no previous transition to GnRH. To improve the predictions of the transitions from AA, we categorised the men reaching AA into eight AA-risk categories corresponding to the eight ADT risk categories (**Supplementary** **Table** **6**). To determine the risk categories after entering AA, we considered this as a missing data problem. More specifically, men entering the AA state following

1. initial AS without previous curative treatment (n=1,613),
2. RP (with or without adjuvant/salvage RT) (n=1,891),
3. RT (n=2,065)
4. WW (n=1,451)

were in separate imputations combined with 7,178 men initially treated with AA in PCBaSe^Traject^. Based on age, time to initiation of GnRH treatment, time to death and cause of death, time to CCI-change and size of CCI change, the AA-risk categories were imputed using chained equation [11]. For men entering the AA-state directly from AS or WW, the GnRH-risk was never lower than indicated by the TNM-stage, PSA-level and Gleason grade group at date of diagnosis. The imputed AA-risk categories for these groups of men are displayed in **Supplementary** **Table** **5**.

(ADT/AS-failure 🡪 AA)

If the model ADT/AS-failure 🡪 GnRH presented above did not indicate that a man entered GnRH, he was considered to enter the AA-state.

(RP 🡪 AA)

The transition probabilities for RP 🡪 GnRH were determined with a logistic regression as specified in **Supplementary** **Table** **6**.

(RT 🡪 AA)

This transition was handled with a logistic regression as specified in **Supplementary** **Table** **6**.

(RT_adj/salv_ 🡪 AA)

This transition was handled using logistic regression as specified in **Supplementary** **Table** **6**.

(WW 🡪 AA)

Since this is an observable transition for men starting in WW, we used a logistic regression model as specified in **Supplementary** **Table** **6**. As for the transition WW 🡪 GnRH, the we used two models.

One for men in risk categories WW_1_-WW_6_, whereas men in risk categories AS_1_-AS_3_ were handled in accordance with our solution for the non-observable transition dates, as previously described [12]. For further details see **Supplementary** **Table** **6**.

(AA 🡪 AA-risk)

Ordinal regression models were used to estimate the probabilities of entering the different AA- risk categories. The ordinal regression models are specified in **Supplementary** **Table** **6**.

**Transitions to RT**

This transient state can only be reached in two ways: either directly or indirectly after a time on AS. In the same manner as for then AA- and GnRH-states, we created RT risk categories to enhance the precision in predictions of further transitions from RT. The RT risk categories were determined by TNM stage, Gleason Grade Groups (GGG) and PSA level at date of diagnosis in accordance with **Supplementary** **Table** **4**. As the TNM stage, GGG and PSA at date of radiotherapy was unknown for the 1,231 men starting on AS, we also included 19,019 men initially treated with RT and with known RT-risk category. We treated the missing RT risk category for men undergoing the transition (AS 🡪 RT) as a missing data problem and used chained equation [11]. **Supplementary** **Table** **6** presents the imputed RT-risk for the 1,231 men starting on AS who later underwent curative RT. The RT-risk for men initially treated with RT was based on initial TNM-stage, Gleason Grade and PSA, as indicated by the specifications of RT-risk categories in **Supplementary** **Table** **6**.

(RT/RP 🡪 RT)

By applying a logistic regression model to all men reaching the state RT/RP, the state considered absorbing when solving the non-observable state transition problem*,* we could determine the probability of entering the transient RT-state. The details for the logistic regression model are specified in **Supplementary** **Table** **6**.

(RT 🡪 RT risk)

For men initially treated with RT, the RT-risk was determined from the TNM-stages, PSA-level and Gleason score at date of diagnosis. Men initially treated with AS were given a RT-risk category based on imputation and an ordinal regression described in **Supplementary** **Table** **6**.

**Transitions to RP**

The state RP could be reached either directly or indirectly following a transition from AS. To enhance the precision in predictions of further transitions from RP, we divided RP into six different RP risk categories based on pathology determined T-stage (pT-stage) and pathology Gleason Grade Group based on radical prostatectomy specimen (pGGG1/ pGGG2/…/ pGGG5). The definition of the six RP-risk categories is shown in **Supplementary** **Table** **3**. RP-risk categories were determined with an ordinal regression model as specified in **Supplementary** **Table** **6**. In case of missing data for pT-stage and pGGG, we used chained equations [11]. **Supplementary** **Table** **3** presents the imputed RP-risk for the 2,347 men starting on AS who later underwent RP.

(RT/RP 🡪 RP)

We used the same model as for the transition RT/RP 🡪 RT above. If a man did not undergo the RT/RP 🡪 RT transition, he was considered to have followed the transition RT/RP 🡪 RP.

(RP 🡪 RP-risk)

Since men entering this state have additional information based on pathological T-stage and pathology Gleason Grade Group, we created a single ordinal regression model for both types of men entering this stage. The model is further specified in **Supplementary** **Table** **3**.

**Transitions to RT_adj/salv_**

This transition was possible for men who were initially treated with RP and for men following the path AS 🡪 RP 🡪 RT_adj/salv_. In total, we found 4,236 men entering this RT_adj/salv_ state. Only 225 men were observed to follow the latter path and the remaining 4,011 followed the path RP 🡪 RT_adj/salv_ in PCBaSe^Traject^.

(RP 🡪 RT_adj/salv_)

The probabilities for these transitions were modelled using logistic regression. The strongest predictor of RT_adj/salv_ following RP was the RP-risk category, which was included in the logistic regression. The complete set of covariates used in the model for this transition is displayed in **Supplementary** **Table** **4**.

*2.3 Transition model specifications*

For each patient, the date of diagnosis was defined as time zero, after which follow-up continued until last day of follow-up, denoted *t*_1_. As ADT was identified in the Prescribed Drug Registry, which started on 1 July, 2005, 1 January 2006 was used as study entry. To allow for longer follow-up, men with a date of diagnosis prior to this date were also included and the follow-up was handled with left truncation.
