# Supplementary material for: How to measure temporal changes in care pathways for chronic diseases using health care registry data
Source: BMC Med Inform Decis Mak. 2019 May 30;19:103. doi: 10.1186/s12911-019-0823-y (PMC6543619; doi:10.1186/s12911-019-0823-y)
Supplement: Supplementary file 2 — Supplementary figures and tables. (DOCX 212 kb) [file 12911_2019_823_MOESM2_ESM.docx]

**Table S1.** Specification of risk categories for men starting active surveillance

| **AS-risk category** |  |
| --- | --- |
| AS_1_ (Favourable low-risk PCa). | T1c or T2,  positive cores ≤ 33%,  cancer length ≤6mm  GGG 1,  PSA<10 ng/ml,  prostate volume < 90cc,  PSA density < 0.2,  6-12 core biopsies |
| AS_2_ (Non-favourable low-risk PCa). | Gleason Score ≤6,  PSA<10 ng/ml,  T1 or T2 with at least one of the following characteristics:   - prostate volume ≥ 90cc, - PSA density ≥ 0.2, - ≤5 or ≥13 core biopsies performed, - positive cores > 33%, - cancer length >6mm |
| AS_3_ (Favourable intermediate risk PCa) | Gleason Score^1^ 3+4  PSA<10 ng/ml,  T1 or T2 |

Men with GGG1 and 10≤PSA≤15 ng/ml were allowed to be In the AS_3_-favourable intermediate risk PCa category.

**Table S2.** Specification of RP-risk categories based on pathological T-stage and pathological Gleason Grade Group.

| **RP risk category** | **pT stage** | **pGGG** | **Imputed RP-risk for men starting in AS,**  **N (%)** |
| --- | --- | --- | --- |
| RP_1_ | T2 or T3a | 1 | 1176 (50.1) |
| RP_2_ | T2 | 2 | 497 (21.2) |
| RP_3_ | T2 | 3-5 | 84 (3.6) |
| RP_4_ | T3a | 2-5 | 434 (18.5) |
| RP_5_ | T3b | 1-5 | 130 (5.5) |
| RP_6_ | T4 | 1-5 | 26 (1.1) |

**Table S3.** Specification of RT risk categories models based TNM-stage, PSA-levels and Gleason Grade Group at date of diagnosis.

| **RT-risk category** | **T stage** | **PSA** | **GGG** | **N stage** | **M stage** | **Imputed RT-risk for men starting in AS,**  **N (%)** | |  |
| --- | --- | --- | --- | --- | --- | --- | --- | --- |
| RT_1_ | T1-2 | PSA<10 | 1 | N0/NX | M0/MX | 198 (16.1) | |  |
| RT_2_ | T1-2 | 10≤PSA<20 | 1 | N0/NX | M0/MX | 97 (7.9) | |  |
| RT_3_ | T1-2 | PSA<10 | 2 | N0/NX | M0/MX | 181 (14.7) | |  |
| RT_4_ | T1-2 | PSA<20 | 3 | N0/NX | M0/MX | 215 (17.5) | |  |
|  | T1-2 | 10≤PSA<20 | 3 | N0/NX | M0/MX |  |  |  |
| RT_5_ | T1-2 | 20≤PSA<50 | 1-3 | N0/NX | M0/MX | 79 (6.4) | |  |
| RT_6_ | T1-2 | PSA<50 | 4 | N0/NX | M0/MX | 229 (18.6) | |  |
|  | T3 | PSA<50 | 1 | N0/NX | M0/MX |  |  |  |
| RT_7_ | T3 | PSA<50 | 2-5 | N0/NX | M0/MX | 129 (10.5) | |  |
| RT_8_ | T1-2 | PSA<50 | 5 | N0/NX | M0/MX | 103 (8.4) | |  |
|  | T4 or 50≤PSA<100 or N1 with any GGG and M0/MX | | | | | |  | |

**Table S4.** Specification of WW risk categories, for men starting in WW but who were not in risk categories AS_1_, AS_2_, or AS_3_, based T-stage, PSA-levels and Gleason Grade Group at date of diagnosis.

| **WW score** | | | | | | | | | | **WW risk categories** | |
| --- | --- | --- | --- | --- | --- | --- | --- | --- | --- | --- | --- |
| **T-stage**  Level Score | | **N-stage**  Level Score | | **M-stage**  Level Score | | **PSA**  Level Score | | **GGG**  Level Score | | **WW-risk category**^&^ | **Imputed WW-risk - N (%)**^#^ |
| T1a | 1 | N0 | 0 | M0 | 0 | PSA≤20 | 1 | 1 | 1 | WW_1_ (Total score≤4) | 1001 (13.7) |
| T1b | 2 |  |  |  |  |  |  | 2 | 2 | WW_2_ (Total score=5) | 1004 (13.8) |
| T1c | 3 | Nx | 0 | Mx | 0 | 20<PSA≤50 | 2 | 3 | 3 | WW_3_ (Total score=6) | 947 (12.9) |
| T2 | 4 |  |  |  |  |  |  | 4 | 4 | WW_4_ (Total score=7) | 1307 (17.9) |
| T3 | 6 | N1 | 7 | M1 | 8 | PSA>50 | 3 | 5 | 5 | WW_5_ (Total score=8) | 1281 (17.6) |
| T4 | 7 |  |  |  |  |  |  | 6 | 6 | WW_6_ (Total score≥5) | 1746 (24.0) |

^#^ For men starting in WW except risk categories AS_1_-AS_3_. Main reason for missing data is fine needle biopsies verifying diagnosis yielding missing GGG.

^&^ Total score calculated as the sum of scores retrieved from T-stage, N-stage, M-stage, PSA, and GGG.

**Table S5.** Specification of ADT- risk categories models based TNM-stage, PSA-levels and Gleason Grade Group at date of diagnosis.

| **ADT-risk group** | **T-stage** | **PSA** | **GGG** | **N-stage** | **M-stage** | **Imputed AA-risk for men** | | | | | | | | **Imputed GnRH-risk for men** | | | | | |
| --- | --- | --- | --- | --- | --- | --- | --- | --- | --- | --- | --- | --- | --- | --- | --- | --- | --- | --- | --- |
|  |  |  |  |  |  | **AS**  **N (%)** | | **primary better? RP,**  **N (%)** | | **Starting**  **in RT,**  **N (%)** | | **Starting**  **in WW,**  **N (%)** | | **starting**  **in AS,**  **N (%)** | | **Starting**  **in RP,**  **N (%)** | **Starting**  **in RT,**  **N (%)** | **Starting**  **in WW,**  **N (%)** | **Starting**  **in AA,**  **N (%)** |
| ADT_1_ | T1-2 | PSA<10 | 1 | N0/NX | M0/MX | 34 (2.1) | | 183 (9.7) | | 163 (7.9) | | 25 (1.7) | | 14 (0.8) | | 46 (3.6) | 72 (3.7) | 21 (1.1) | 34 (1.9) |
| ADT2 | T1-2 | 10≤PSA<20 | 1 | N0/NX | M0/MX | 125 (7.7) | | 171 (9.0) | | 206 (10.0) | | 87 (6.0) | | 44 (2.5) | | 64 (4.9) | 101 (5.2) | 111 (5.8) | 91 (5.1) |
| ADT_3_ | T1-2 | PSA<10 | 2-3 | N0/NX | M0/MX | 125 (7.7) | | 366 (19.4) | | 380 (18.4) | | 312 (21.5) | | 55 (3.2) | | 101 (7.8) | 155 (8.0) | 189 (9.9) | 134 (7.6) |
| ADT_4_ | T1-2 | 10≤PSA<20 | 2-3 | N0/NX | M0/MX | 225 (13.9) | | 287 (15.2) | | 322 (15.6) | | 270 (18.6) | | 108 (6.2) | | 56 (4.3) | 95 (4.9) | 168 (8.8) | 89 (5.0) |
| ADT_5_ | T1-2 | PSA<50 | 4-5 | N0/NX | M0/MX | 212 (13.1) | | 304 (16.1) | | 333 (16.1) | | 289 (19.9) | | 146 (8.4) | | 136 (10.5) | 220 (11.4) | 296 (15.5) | 207 (11.7) |
| ADT_6_ | T3 | PSA<50 | 1-5 | N0/NX | M0/MX | 291 (18.0) | | 161 (8.5) | | 182 (8.8) | | 192 (13.2) | | 299 (17.3) | | 130 (10.1) | 164 (8.5) | 327 (17.1) | 189 (10.7) |
| ADT_7_ | T4 or 50≤PSA<100 or N1 with GGG 1-5 and M0/MX | | | | | | 318 (19.7) | | 157 (8.3) | | 150 (7.3) | | 139 (9.6) | | 318 (19.7) | 115 (8.9) | 262 (13.5) | 335 (17.5) | 224 (12.7) |
| ADT_8_ | M1 or PSA≥100 | | | | | | 283 (17.5) | | 262 (13.9) | | 329 (15.9) | | 137 (9.4) | | 283 (17.5) | 645 (49.9) | 866 (44.8) | 463 (24.2) | 800 (45.2) |

**Table S6.** Specification of models used to estimate transition probabilities from observable states as described in Figure 1.

| **Transition** | **Model** | **Covariates** | **Covariates details** |
| --- | --- | --- | --- |
| **AS → death,**  **WW → death,**  **RP → death,**  **RT → death,**  **RT_adj/salv_ → death,**  **AA → death,**  **and**  **GnRH → death** | Logistic regression | Age, CCI, first management strategy, time since curative treatment/ADT | Age as a linear term for men < 70 and as a quadratic term for men ≥ 70.  CCI as a linear term. Interaction between age and CCI. Current state (GnRH following curative treatment, GnRH following AS/WW, AA following curative treatment, AA following AS/WW, AS, WW, RP, RT). Interaction between time after curative treatment, age, comorbidities, current state. |
| **AA → PCa-death** | Logistic regression | Age, CCI, AA-risk category, path taken to reach the state AA | Age (continuous).  CCI (0/1/2/3/4+).  AA-risk category (eight levels).  Path (Primary AA / AA after WW / AA as first treatment after AS / AA following curative therapy). |
| **GnRH → PCa-death** | Logistic regression | Age, CCI, GnRH-risk category, Time in state | Age (continuous).  CCI (0/1/2/3/4+).  GnRH-risk category (eight levels).  Time in state as a linear term, and time in state < 1 month (Yes/No). |
| **RT/RP → RP**  **and**  **RT/RP → RT**  (for AS treated men) | Logistic regression | Age, CCI, time in AS, PSA at DX, risk category | Age (continuous). CCI (0/1/2/3+). Time in AS (continuous). PSA at DX considered as a constant for PSA<3 and linear in the range 3-15. Risk category (AS_1_/ AS_2_/ AS_3_). |
| **RP → RP-risk** | Ordinal regression | Age, CCI, time in AS, PSA at DX, risk category | Age (continuous).  CCI (0/1/2/3+).  Time in AS (continuous).  PSA at DX considered as a constant for PSA<3 and linear in the range 3-15.  Risk category (AS_1_/ AS_2_/ AS_3_). |
| **RT → RT-risk** | Ordinal regression | Age, CCI, time in AS, PSA at DX, risk category | Age (continuous).  CCI (0/1/2/3+).  Time in AS (continuous).  PSA at DX considered as a constant for PSA<3 and linear in the range 3-15.  Risk category (AS_1_/ AS_2_/ AS_3_). |
| **RP → RT_adj/salv_** | Logistic regression | Age, CCI, RP-risk category, time spent in the RP state, Path taken to the RP state | Age continuous.  CCI (0/1/2/3/4+).  RP-risk category (6 levels).  Time spent in the RP state (linear). Interaction between time in RP state and risk category (constant between 0-0.5 years, linear between 0.5-2 years, 0 above 2 years for risk categories 4-6).  Path taken to the RP state (RP direct/RP after AS). |
| **RP → AA** | Logistic regression | Age, CCI, RP-risk category, time spent in the RP state, Path taken to the RP state | Age (linear with and a quadratic term for men ≥80).  CCI (0/1/2/3/4+).  RP-risk category (6 levels).  Time spent in the RP state (continuous).  Path taken to the RP state (RP direct/RP after AS). |
| **AA → AA-risk**  (from RP or RT_adj/salv_) | Ordinal regression | Age, CCI, RP-risk category, time spent in the RP state, Path taken to the AA state | Age (continuous).  CCI (0/1/2/3/4+).  RP-risk category (6 levels).  Time spent in the RP/RT_adj/salv_-state (continuous).  Path taken to the AA-state (Direct RP / Direct from RP following AS / RT_adj/salv_ following direct RP / RT_adj/salv_ following RP after AS). |
| **RP → GnRH** | Logistic regression | Age, CCI, RP-risk category, time spent in the RP state, Path taken to the RP state | Age (continuous).  CCI (0/1/2/3/4+).  RP-risk category (6 levels).  Time spent in the RP state (continuous).  Path taken to the RP state (RP direct/RP after AS). |
| **GnRH → GnRH-risk**  (from RP or RT_adj/salv_) | Ordinal regression | Age, CCI, RP-risk category, time spent in the RP state, Path taken to the GnRH state | Age (continuous).  CCI (0/1/2/3/4+).  RP-risk category (6 levels).  Time spent in the RP/RT_adj/salv_-state (continuous).  Path taken to the GnRH-state (Direct RP / Direct from RP following AS / RT_adj/salv_ following direct RP / RT_adj/salv_ following RP after AS). |
| **RT → AA** | Logistic regression | Age, CCI, RT-risk category, Path taken to the RT state | Age (continuous).  CCI (0/1/2/3/4+).  RT-risk category (8 levels).  Path taken to the RT-state (RT direct/RT after AS). |
| **AA → AA-risk**  (from RT) | Ordinal regression | Age, CCI, RT-risk category, Time spent in the RT state | Age (continuous).  CCI (0/1/2/3/4+),  RT-risk category (8 levels),  Time spent in the RT state (continuous). |
| **RT → GnRH** | Logistic regression | Age, CCI, RT-risk category, Path taken to the RT state, Time spent in RT state | Age (continuous).  CCI (0/1/2/3/4+).  RT-risk category (8 levels).  Path taken to the RT-state (RT direct/RT after AS).  Time spent in RT state (continuous). |
| **GnRH → GnRH-risk**  (from RT) | Ordinal regression | Age, CCI, RT-risk category, Time spent in the RT state | Age (continuous).  CCI (0/1/2/3/4+).  RT-risk category (8 levels).  Time spent in the RT state (continuous). |
| **RT_adj/salv_ → AA** | Logistic regression | Age, CCI, RP-risk category, Path taken to the RT**_adj/salv_** state | Age (continuous).  CCI (0/1/2/3/4+).  RP-risk category (6 levels).  Path taken to the RT**_adj/salv_** -state (RP direct/RP after AS). |
| **RT_adj/salv_ → GnRH** | Logistic regression | Age, CCI, RP-risk category, Time spent in the RT**_adj/salv_**-state, Path taken to the RT**_adj/salv_** state | Age (continuous).  CCI (0/1/2/3/4+).  RP-risk category (6 levels).  Time spent in the RT**_adj/salv_**-state (continuous). Path taken to the RT**_adj/salv_** -state (RP direct/RP after AS). |
| **WW → AA**  (for men initially treated with WW) | Logistic regression | Age, CCI, WW-risk category, Time spent in the WW-state | Age (continuous).  CCI (0/1/2/3/4+).  WW-risk category (6 levels).  Time spent in the WW-state (continuous). |
| **WW → GnRH**  (for men initially treated with WW) | Logistic regression | Age, CCI, WW-risk category, Time spent in the WW-state | Age (continuous).  CCI (0/1/2/3/4+).  WW-risk category (6 levels).  Time spent in the WW-state (continuous). |
| **AA → AA-risk**  (from **WW**) | Ordinal regression | Age, CCI, WW-risk category, Time spent in the WW state | Age (continuous).  CCI (0/1/2+).  WW-risk category (6 levels),  Time spent in the WW state (continuous). |
| **GnRH → GnRH -risk**  (from **WW)** | Ordinal regression | Age, CCI, WW-risk category, Time spent in the WW state | Age (continuous).  CCI (0/1/2+).  WW-risk category (6 levels).  Time spent in the WW state (continuous). |
| **AA → GnRH** | Logistic regression | Age, CCI, AA-risk category, Path taken to reach the AA-state, Time spent in the AA state | Age (continuous).  CCI (0/1/2/3/4+).  AA-risk category (8 levels).  Path (Primary AA / AA after WW / AA as first treatment after AS / AA following curative therapy). Time spent in the AA state (continuous). |
| **GnRH → GnRH-risk**  (from AA) | Ordinal regression | Age, CCI, AA-risk category, Time spent in the AA state, Path taken to reach the AA-state | Age (continuous).  CCI (0/1/2+).  AA-risk category (8 levels).  Time spent in the AA state (continuous).  Path (Primary AA / AA after WW / AA as first treatment after AS / AA following curative therapy) |

**Figure S1:** Simplified (i) and detailed (ii) graphical representation of state-transition model used to handle the state androgen deprivation therapy ADT and it’s descendant states. Rather than considering all types of ADT together we split it into anti androgen monotherapy (AA) and GnRH treatment (including orchiectomy).

**ADT**

**Death**

**PCa**

**death**

**CCI**

**GnRH**

**Death**

**PCa**

**death**

**CCI**

**AA**

**CCI**

**(i)**

**(ii)**

ADT risk category 1

ADT risk category 8

**Figure S2:** Graphical representation of state-transition model used to estimate non- observable transition probabilities including: active surveillance (AS) curative treatment (radical prostatectomy/radiotherapy; RP/RT), death, AS failure, or watchful waiting (WW), and androgen deprivation therapy (ADT). AS and WW circles represent transient stages and orange circles represent absorbing states. The blue and green circles represent additional information gathered to facilitate the estimation of transition probabilities (Biopsy and Charlson Comorbidity Index (CCI)).

AS_3_

AS_2_

**ADT**

**AS**

**failure**

**Death**

**AS**

**WW**

**CCI**

**CCI**

**Biopsy**

**RP/RT**

**AS**

**CCI**

**Biopsy**

AS_1_

**Figure S3:** The watchful waiting state is modelled as two distinct states WW_AS_ and WW_Non-AS_. Men satisfying the AS_1_, AS_2_, and AS_3_ criteria but were recorded as WW in NPCR were modelled together with men starting in AS and later switch to WW considering the three AS risk categories. Men not candidates for AS were categorised into six risk categories WW_1_, WW_2_, …, WW_6_. No transitions between WW_AS_ and WW_Non-AS_ were considered.

**WW_Non-AS_**

**WW_AS_**

**Biopsy**

**CCI**

AS_1_-risk

**CCI**

AS_2_-risk

AS_3_-risk

WW_1_-risk

WW_2_-risk

WW_3_-risk

WW_4_-risk

WW_5_-risk

WW_6_-risk

**AS**

**Biopsy**

**CCI**
